# Indonesian market demand patterns for food commodity sources of carbohydrates in facing the global food crisis

**DOI:** 10.1016/j.heliyon.2023.e16809

**Published:** 2023-05-30

**Authors:** Fachrur Rozi, Agung Budi Santoso, I Gusti Ayu Putu Mahendri, Ronald Timbul Pardamean Hutapea, Demas Wamaer, Viktor Siagian, Dian Adi Anggraeni Elisabeth, Sugiono Sugiono, Handoko Handoko, Herman Subagio, Amiruddin Syam

**Affiliations:** a Research Center for Macroeconomics and Finance, National Research and Innovation Agency Republic of Indonesia - BRIN, Jakarta 12710, Indonesia; b Research Center for Behavioral and Circular Economics, National Research and Innovation Agency Republic of Indonesia - BRIN, Jakarta 12710, Indonesia; c Research Center for Horticultural and Estate Crops, National Research and Innovation Agency Republic of Indonesia – BRIN, Cibinong, Bogor Regency 16915, Indonesia

**Keywords:** Crisis, Demand pattern, Food, Global

## Abstract

Global food consumption tends to rise more quickly than supply. This has to do with important global issues like population growth. Additionally, global conflicts are going to hinder the distribution of food. Indonesia has an enormous opportunity of anticipating these circumstances considering its promise as one of the largest supplies of food worldwide. Rice is still the staple food in Indonesia, but the dynamics of society are under threat from wheat food. It is possible to create strategy plans to deal with potential food scarcity by understanding the behavior of food demand trends for big carbohydrate sources like corn, cassava, sweet potatoes (as a substitution), and the development of wheat as a beneficial food. The results of the study indicate that rice, corn, cassava, and sweet potatoes—food commodities that are major sources of carbohydrates—are inelastic, which means that their prices are not affected by variations in demand. The community still relies on rice as the primary food source. Cross elasticity >0 in these non-wheat food commodities indicates mutually beneficial replacement among the foods that are sources of carbohydrates. That is, with the dynamics of an increase in income, for example, it will also increase consumption. The results of the study also demonstrate that wheat food items are only a complementary, not a staple food needed, thus concerns about wheat's dominance as a food component in industrial products actually have no impact on local food. The availability of high-yielding varieties of rice, corn, cassava, and sweet potatoes, the implementation of food reserves by the Indonesian National Logistics Agency (*Bulog*) from the government center to the regions, food diversification, changing preferences, and creating an awareness of local food pride with massive education are some of the anticipatory steps taken in response to the global food crisis.

## 1 Introduction

According to the Global Food Crisis (GFC), hunger levels worldwide remain extremely high in 2021. In 53 countries, around 193 million people are severely under severe food insecurity and urgently need help. This is an increase of nearly 40 million people over the previous year's total. The conflict in Ukraine is likely to exacerbate the severe escalation of food insecurity predicted for 2022. Project analysis of the impact of the conflict on global food, energy, fertilizer price, and supplies have been inadequate at most country levels [1].

Food demand in the world tends to rise faster than supply. Food and Agriculture Organization (FAO) predicts that food needs for developing countries will rise by 60% in 2030 and double by 2050, equivalent to the need to increase world production by 42% in 2030 and 70% in 2050 [2,3]. According to the prediction, food consumption will rise as the world population grows by 73 million annually between 1995 and 2020. This equals a 32% rise, with the world's population expected to reach 7.5 billion in 2020 [4].

Food demand has risen significantly as the world's population, wealth, and lifestyle have increased. Meanwhile, commodity production is increasing as food supplies fail to meet demand. As a result, food security is becoming increasingly important [5,6]. Food security is a worldwide concern and the century's greatest challenge. Each country has different investment methods and support to guarantee food security [7]. Global food costs have risen significantly year after year [3]. The trend of world grain production has decreased significantly. In 2014–2015, global grain production reached 2,514 million tons, with the United States, China, India, and Brazil producing the most grain. However, global grain production fell to 2,456 million tons in 2015–2016. Global climate change, decreasing productive agricultural area, and soil fertility are the primary causes of the global grain production drop [8,9,10,11,12].

Food availability must be equal to or greater than the population's food demand. The supply of sufficient food in a particular location (market) does not guarantee the availability of food at the home level since it is dependent on families' capability to obtain food both physically (reaching power) and economically (purchasing power) [13,14]. As a result, food security guarantees food availability at the individual level. The supply of sufficient, diversified, nutritious, and balanced food, both quantity and quality, is essential for developing a country's human resources. Food insecurity can cause unrest and influence social, security, and economic issues [15,16].

The commodity resource of carbohydrates that people in Indonesia are still the most invested in is rice. Rice, flour, grains, and tubers are considering as common products, whereas bread and prepared food are considered as luxury foods [17]. The Ministry of Agriculture's Center for Agricultural Data and Information Systems (Pusdatin) reported that rice usage per capita fell from 86.82 kg in 2017 to 81.60 kg in 2018 [18]. The data demonstrated an alteration in the trend of carbohydrate food intake away from rice and toward other foods with carbohydrate sources [19]. In a research report, the United States Department of Agriculture (USDA) predicted that Indonesia will surpass the United States as the world's top importer of wheat in 2017–2018, with an amount of approximately 12.5 million tons. This increase was because as the population increased, so did the desire for food. Additionally, the increase in wealth was also a factor in the increase in wheat flour shipments [20].

Indonesia has an enormous opportunity of producing food utilizing other carbohydrate sources in the context of food security. Using local food items as a supply of carbohydrates depends on their ability to enhance the community's health. In our system of food security, corn, cassava, and sweet potatoes are significant sources of carbohydrates. These commodities have excellent chances to replace rice and can be turned into flour to replace imported wheat flour [21,22].

However, changing people's food consumption patterns is not easy. Changes in preferences, prices, income, food prices for other connected carbohydrate sources, the number of household members, age and gender of the household head, and work activities can lead to the changes in eating habits. Every year, the amount of rice consumed at home is getting smaller and replaced with ready-to-eat foods due to the hectic lifestyle of the people [18]. Food preferences and eating habits change on a regular basis. Prices will change either up or down, which will affect preferences. According to the supply and demand law, customers usually reduce their consumption of products when the prices rise and vice versa. The type of foods that people favor also depends on their income. Households with high income will have a wider preference for food sources of carbohydrates compared to the low-income households. Due to their limited resources, low-income households do not have many options to change their eating habits [23].

Food diversity is the process of increasing the consumption of a range of foods while adhering to the idea of getting enough food. The lack of substitute products that are easy to find and affordable by the community is one of the obstacles in developing local food. In addition, the assumption that rice is the only staple food should be considered as the obstacle to food diversity. Utilization of manufacturing and refining technology in local food-based food production is one method to create local food products [24,25].

The aim of the study is to determine consumer behavior towards demand patterns for food sources of carbohydrates in Indonesia. This due to the changes in people's lifestyles and increased income [26,27,28]. Problems appear when there is an increase in the demand for wheat-based foods while people's interest in local-based food products decreases. Dependence on imported food can lead to food instability if the government is unable to provide it, in addition to requiring foreign currency. This may occur as a result of both internal (such as a decrease in foreign currency) and external (such as limited availability in global market) variables. The severity of food insecurity will differ depending on where it occurs, as there may not be a wide variety of food sources or regulations [29,30].

Research on customer behavior in food consumption patterns should be carried out periodically. This is because customer preferences are constantly changing as a result of changing economic capabilities and public awareness of the advantages of food for leading a healthy and fulfilling life. It is crucial to analyze food consumption for carbohydrate sources in order to collect data for making policies that address the food situation in the future. With regards to the predictions of food scarcity, this study seeks to examine the desire for food sources of carbohydrates on a nationwide level.

## 2 Materials and methods

### 2.1 Theoretical framework

Demand is the overall relationship between the amount of certain goods or services that consumers will purchase during a certain period and the price [31]. If prices vary while other factors such as prices of substitute or complementary goods, income, and preferences are considered constants, then the relationship between price and the amount of goods or services that consumers will purchase can be described in a curve obtained from the consumer's equilibrium points. Many factors, including self-price, income, the price of similar items, income distribution, population size, preferences, and other elements, determine the large number of commodities that consumers are willing to buy during a particular time.

By assuming that the variables influencing other variables exist, it is possible to evaluate each variable separately. According to the law of demand, the quantity demanded and the price of goods are inversely related. This implies that demand will increase when the price of the commodity is low, and vice versa. As a result, both price and quantity demanded can be represented by a curve with a negative slope.

Food demand is affected by population in two ways. First, demand increases linearly with population. Second, because population growth can sometimes slow growth in per capita income, the per capita income, which is calculated as total income divided by population, does not always increase demand. In rare cases, where income does not increase at all, per capita income increases as population grows. This circumstance can counteract the direct effect of population growth on food demand [31].

### 2.2 Data collection technique

The time series data used in this study is secondary data which was documented in the period of 2007 to 2020 and was obtained from FAOSTAT, Statistics Indonesia (BPS), and The Ministry of Agriculture's Center for Agricultural Data and Information Systems (Pusdatin). Collecting and confirming data from FAOSTAT at the following links: https://www.fao.org/faostat/en/#data, https://www.fao.org/statistics/en/, and https://www.fao.org/food-agriculture-statistics/en/are data collection steps. In addition, FAOSTAT data from databases at the BPS and the Ministry of Agriculture will be supplemented for the desk research databases at the BPS and the Ministry of Agriculture. The institutional links are https://satudata.pertanian.go.id/, https://www.bps.go.id/subject/5/konsumsi-dan-pengeluaran.html, and https://www.bps.go.id/.

In this study, data on Indonesia's per capita income, food needs, and commodity prices were collected for analysis. From the three data collected, it is possible to obtain the annual average statistics for the general population in Indonesia, both in rural and urban areas. Five food commodity sources of carbohydrates namely corn, cassava, sweet potatoes, wheat, and rice were used as comparison. Since FAO does not provide a separate price variable, the price for each food commodity is calculated by dividing household expenditure (IDR) by the total amount of food purchased. The amount spent was for the food groups of tubers, grains, rice, and flour. The Ministry of Agriculture and BPS provided each of these supporting databases in specific details.

### 2.3 Data analysis method

1 Exponential smoothing technique for using time series data

The data investigated in this study cover the years 2007 to 2020, so that by using the exponential smoothing technique, a logical price was obtain for the same time period in one unit of observation [32]. By employing an exponential smoothing technique will reduce the bias in the use of time series data in the Log-Linear model. To see the overall long-term flow of data, time series data can be smoothed using the exponential smoothing approach. For example, data on the real price of a commodity for a specific time period can be obtained by using exponential smoothing to generate long-term predictions (one or two periods in the future) for a time series. This method provides a series of exponentially weighted moving averages over a time series, that is, throughout the series, any calculation or forecast of future smoothing depends on all observed values that precede it. The values of the most recent observation are given the highest weight in exponential smoothing, followed by the values of the previous observation, and so on, with the value of the earliest observation getting the smallest weight [33]. So, the general equation in the exponential smoothing model is:

$$Y_{t+1}={aX}_{t}{+\left(1-a\right)Y}_{t}$$

where:

*X*_*t*_ = the actual value
*Y*_*t*_ = the last prediction
*Y*_*t+1*_ = prediction for the next period
*a* = smoothing constant

The exponential smoothing technique approach was applied in this study's time series data processing using MINITAB and SPSS software [34].

2 The demand function model for food commodities

In this study, the data were analyzed using the theory of the relationship between the elasticity coefficients in the demand elasticity matrix. Pyles describes the method of estimating elasticity with this matrix in “Demand Theory and Elasticity Matrix Construction” [35]. This method is more efficient because it can indirectly estimate the elasticity value of a commodity from the income elasticity value. The method can then be applied to situations with limited. Since the regression method involves a limited number of independent variables, multicollinearity is also minimized. In addition, the price variable so far has a tendency for high collinearity [35].

An econometric technique was used to evaluate data from FAOSTAT, using the Double Log, Log-Linear, or Constant-Elasticity Models [36]. Because the regression coefficient of the linear function model directly reflects the elasticity coefficient of each variable [37], it is used to estimate the elasticity of demand for food sources of carbohydrates in Indonesia. As demonstrated by Refs. [[37], [38], [39]], this model has long been used to study food consumption patterns.

The demand for a commodity is certainly influenced by many factors simultaneously. In simple terms [36], explained that in purchasing a number of commodities i, a consumer will definitely be influenced by the price of the commodity (p) and the total income (I) (as an income approach) with the function equation as follows:

qi=gi(I,p)

The above function is called “Marshallian demand function.” Several other factors affecting demand include prices of other commodities, market taste, income distribution, population, consumer welfare, and government policies. In traditional demand theory, the factors that affect demand are emphasized on four items, namely the price of the commodity in question, the prices of other commodities, consumer income, and tastes. The Marshallian demand function derives price elasticity, cross elasticity, and income elasticity [40]. This is indicated by the following formula:

$$e_{ii}=1+\left(\frac{\gamma_{ij}}{W_{j}}\right)-\beta_{i}$$

Price elasticity

Cross elasticity

$$e_{ii}=\left(\frac{\gamma_{ij}}{W_{j}}\right)-\beta_{i}\left(\frac{W_{j}}{W_{j}}\right)$$

Income elasticity

$$\eta_{i}=1+\left(\frac{\beta_{i}}{W_{i}}\right)$$

The technique for estimating the coefficients of the linear regression equations in the linier log model is Ordinary Least Squares (OLS). The OLS Estimator makes it easy to use this model to obtain the best estimate. The BLUE (Best Linear Unbiased Estimator) characteristics are generated in manufacture of OLS estimators. Real food prices (P), level of demand (Q) for the five commodities studied, and real income (I) per person for the entire population are the variables considered. The exponential model must first be transformed as shown below so that the non-linear model becomes a linear model [40,41]:

$$Q={\alpha \;P^{\beta }\;I}^{\beta }$$

The natural logarithm equation can be transformed as shown below to estimate the previously mentioned equation:

lnQ=α+β1lnP+β2lnI+e

The demand function model for each food commodity:

Rice commodity: Q_rice_ = α P^β^_rice_ I^β^_rice_

Corn commodity: Q_corn_ = α P^β^_corn_ I^β^_corn_

Cassava commodity: Q_cassava_ = α P^β^_cassava_ I^β^_cassava_

Sweet potato commodity: Q_sweet potato_ = α P^β^_sweet potato_ I^β^_sweet potato_

Wheat commodity: Q_wheat_ = α P^β^_wheat_ I^β^_wheat_

The demand function model for food commodities correlation:

Rice to corn, cassava, sweet potato, wheat:

Q_rice_ = α P^β^_corn_ Q_rice_ = α P^β^_cassava_ Q_rice_ = α P^β^_sweet potato_ Q_rice_ = α P^β^_wheat_

Corn to rice, cassava, sweet potato, and wheat:

Q_corn_ = α P^β^_rice_ Q_corn_ = α P^β^_cassava_ Q_corn_ = α P^β^_sweet potato_ Q_corn_ = αP^β^_wheat_

Cassava to rice, corn, sweet potato, and wheat:

Q_cassava_ = α P^β^_rice_ Q_cassava_ = α P^β^_corn_ Q_cassava_ = α P^β^_sweet potato_ Q_cassava_ = αP^β^_wheat_

Sweet potato to rice, corn, cassava, and wheat:

Q_sweet potato_ = α P^β^_rice_ Q_sweet potato_ = α P^β^_corn_ Q_sweet potato_ = α P^β^_cassava_

Q_sweet potato_ = α P^β^_wheat_

Wheat to rice, corn, cassava, and sweet potato:

Q_wheat_ = α P^β^_rice_ Q_wheat_ = α P^β^_corn_ Q_wheat_ = α P^β^_cassava_ Q_wheat_ = α P^β^_sweet potato_,

where:

*Q* = Need of food commodities quantity for rice, corn, cassava, sweet potato, and wheat per capita per year (kg)
*P =* Price of each food commodity for rice, corn, cassava, sweet potato and wheat (IDR/kg)
I *=* Income per capita per year (IDR)
Β = Coefficient elasticity of each commodity for rice, corn, cassava, sweet potato and wheat
α = Constant of demand function

Similar to the theory of Pyles (1989), the elasticity values of many of the analyzed commodities can be calculated using this demand elasticity matrix approach by simply determining the elasticity value itself or the cross-elasticity value of one of the commodities and the income elasticity value of product. This will determine the elasticity of one of the commodities and the value of the income elasticity of the five commodities using the findings of the regression of the demand functions for the four commodities [42].

## 3 Results and discussion

### 3.1 Food consumption pattern of the community

In Indonesia, the pattern of community food consumption remains dominant in the rice staple food. Rice has become the primary and first staple food. Even people who used to eat non-rice staple foods have switched to rice [43]. Rice and wheat are the most widely consumed foods in urban areas across all economic levels (including their derivatives). Meanwhile, the first staple food pattern for all expenditure groups in rural areas was rice, followed by corn, cassava, and wheat in the low-income group. Meanwhile, in the middle and upper classes, rice was followed by only flour [44,45].

## 4 The elasticity of food demand for carbohydrate sources

These food commodities' prices significantly impact community behavior regarding food consumption patterns. The elasticity value indicates the sensitivity between demand for food and the price level of these food commodities [46]. The elasticity of demand occurs when the price of goods or services affects consumer demand [36]. Consumers will buy more if the price falls. If the price rises, they will stop their purchases and wait for the price to return to normal. The price elasticity of demand will show that the food commodity's behavior is elastic (>1), elastic per unit (=1), and inelastic (<1) [42,47]. The impact of changes in prices of goods, income, and prices of other related goods, whether substitute or complementary, will have different impacts for each commodity [48]. The impact will differ both nationally and within a region. Therefore, it is important to understand the sensitivity of demand for carbohydrate-source food commodities to these changes. This sensitivity can be measured using own-price elasticity, cross-price elasticity, and expenditure/income elasticity [48,49].

Based on the findings of the study, the demand for food commodities in the pattern of the community's behavior, as represented by the value of the elasticity of demand for each carbohydrate food source, is as follows:

### 4.1 Rice commodity

Rice consumption per capita decreased significantly from 2008 to 2020. Meanwhile, per capita consumption of wheat-based foods has risen. Rice is a staple food that all Indonesian households have consumed for generations. Rice consumption at the national level was 93.44 kg per capita per year in 2008, then decreased by 4.24% throughout the year [50]. The average consumption of rice by the Indonesian population has increased since the pandemic, according to research by the [50]. Rice consumption, including local, superior quality, and imported rice, averaged 1.404 kg per capita per week in 2018. In 2019, this number dropped to 1.374 kg per capita per week. When the pandemic struck, however, the average consumption increased to 1.379 kg per capita per week. Consumption has also risen in the second year of the pandemic, reaching 1.451 kg per capita per week in 2021.

The analysis results show that rice's price elasticity is 0.26 (Table 1). This shows that commodity demand is inelastic or unresponsive to price fluctuations. When rice costs rise by 10%, the quantity demanded falls by less than 10%. Meanwhile, the income elasticity of rice demand is 0.24 > 0 (positive). This shows rice as a normal good, indicating that as income increases, so will rice consumption. The conclusion is that rice is still needed by society in general. This indicates that rice is still a staple food in the community. These findings align with prior research that rice was still the most favorable carbohydrate source for Indonesian people. Bread and processed food were considered luxurious, while rice, wheat flour, cereals, and roots were normal goods [17].

**Table 1:** Model of rice demand function on the prices of other food commodities.

Commodities	Demand function model for rice commodity to other crops	Demand elasticity Coefficient (Є)
Rice	Q_rice_ = 7.99 P^0.26^_rice_ I^0.24^_rice_	ЄP_rice_ = 0.26*; ЄI_rice_ = 0.24**
Corn	Q_rice_ = 3.95 P^1^^.^^03^_corn_	1.03**
Cassava	Q_rice_ = 11.11 P^0^^.^^16^_cassava_	0.16**
Sweet potato	Q_rice_ = 10.87 P^0^^.^^18^_sweet potato_	0.18**
Wheat	Q_rice_ = 7.17 P^0^^.^^58^_wheat_	0.58**

Notes: * = significant effect at α level of 10%.

** = significant effect at α level of 5%.

All food commodities analyzed are substitutes for rice. This is indicated by the cross-elasticity value of all commodities analyzed, which is > 0. This means that these foods have the same role or function as rice and can be a substitute food when rice costs rise (Table 1).

### 4.2 Corn commodity

Corn is a normal good (ЄPcorn = 5.09 > 0) and is the most elastic in terms of demand among food commodities. This is indicated by the own-price elasticity of 5.09 > 1. The correlation to the price of food sources of other carbohydrates has a significant effect, as shown by the cross-elasticity (ЄP), which has a positive value of more than 1. This means that even though the price of other foods has increased, it has not changed the increase in demand for corn. Likewise, if people's income increases, the amount of corn demand also, although the effect is insignificant (ЄIcorn = 0.13) (Table 2). This shows that the increase in demand for corn is not due to food consumption but demand for many non-foods (feed) industries.

**Table 2:** Model of corn demand function on the prices of other food commodities.

Commodities	Demand function model for corn commodity to other crops	Demand elasticity Coefficient (Є)
Corn	Q_corn_ = - 30.10 P^5.09^_corn_ I^0.13^_corn_	ЄP_corn_ = 5.09*; ЄI_corn_ = 0.13
Rice	Q_corn_ = −16.97 P^3^^.^^1^^0^_rice_	3.10**
Cassava	Q_corn_ = 3.95 P^1^^.^^0^^0^_cassava_	1.00**
Sweet potato	Q_corn_ = 3.27 P^1^^.^^03^_sweet potato_	1.03**
Wheat	Q_corn_ = −13.53 P^2^^.^^81^_wheat_	2.81**

Notes: * = significant effect at α level of 10%.

** = significant effect at α level of 5%.

The increasing population and better income of Indonesian people have caused an increasing demand for livestock products, especially chicken and eggs. These demands have driven a dramatic increase in feed demand and corn as the main component of feed [51].

### 4.3 Cassava commodity

The analysis shows that cassava's elasticity is positive, so cassava is a normal good and inelastic substitute for other foods (ЄPcassava = 0.62 > 0 biggest than others). The effect of increasing cassava prices is very significant on its demand (ЄPcassava = 0.62**). At the same Time, the increase in income has no significant effect on the demand for cassava (Table 3). So far, the demand for cassava has continued to increase for consumption, animal feed, the processed industry (dried cassava, chips, tapioca, and cassava flour), and new renewable energy materials.

**Table 3:** Model of cassava demand function on the prices of other food commodities.

Commodities	Demand function model for cassava commodity to other crops	Demand elasticity Coefficient (Є)
Cassava	Q_cassava_ = 3.07 P^0.62^_cassava_ I^0.51^_cassava_	ЄP_cassava_ = 0.62**; ЄI_cassava_ = 0.51
Rice	Q_cassava_ = −14.24 P^2^^.^^84^_rice_	2.84**
Corn	Q_cassava_ = −24.28 P^4^^.^^31^_corn_	4.31**
Sweet potato	Q_cassava_ = 3.95 P^1^^.^^0^^0^_sweet potato_	1.00**
Wheat	Q_cassava_ = −12.47 P^2^^.^^73^_wheat_	2.73**

Notes: * = significant effect at α level of 10%.

** = significant effect at α level of 5%.

Cassava is a rice substitute that is important in supporting the food security of regions in Indonesia. However, many obstacles still exist in changing the community's consumption patterns. Therefore, regarding food security in regions, it is necessary to disseminate cassava-based food diversification as an alternative to rice or corn. Various cassava-based products (intermediate and end-products) have been produced in small-scale industries with simple equipment and large-scale with modern machinery [52]. As an intermediate cassava product, tapioca has been growing rapidly in Indonesia. In recent years, modified cassava flour (mocaf) agro-industry has also been started [53]. Several agro-industries produce cassava end-products, such as cakes, chips, brownies, traditional sweets (*dodol*), fermented cassava (*tape* or tapai) and so on. In addition, cassava processing wastes or by-products can be processed into fertilizer, especially for plantation crops, and cassava peels can be processed into animal feed [53].

### 4.4 Sweet potato commodity

The characteristics and behavior of consumers towards sweet potato commodities market are the same as for cassava commodities, namely as normal and inelastic goods, and it can be used as substitutes (ЄP>0) for other foods (Table 4). The demand for sweet potatoes also increases with the need for raw materials for the food processing industry (sauce, snacks, and other functional foods).

**Table 4:** Model of sweet potato demand function on the prices of other food commodities.

Commodities	Demand function model for sweet potato commodity to other crops	Demand elasticity Coefficient (Є)
Sweet potato	Q_sweet potato_ = 10.32 P^0.16^_sweet potato_ I^0.15^_sweet potato_	ЄP_sweet potato_ = 0.16; ЄI_sweet potato_ = 0.15
Rice	Q_sweet potato_ = 7.85 P^0.54^_rice_	0.54**
Corn	Q_sweet potato_ = 2.76 P^1.2^^^1^^_corn_	1.21**
Cassava	Q_sweet potato_ = 11.36 P^0.19^_cassava_	0.19**
Wheat	Q_sweet potato_ = 3.95 P^1.00^_wheat_	1.00**

Notes: * = significant effect at α level of 10%.

** = significant effect at α level of 5%.

The dependence and linkages of the demand for commodities provide different consumer behavior patterns in viewing a product. Information on the development of alternative sweet potato foods in terms of demand elasticity is needed. Through this analysis, people's behavior and consumption patterns will be known due to the influence of changes in people's income (income elasticity) and the price level of its and other commodities (own and cross elasticity) of sweet potato demand [54,55].

### 4.5 Wheat commodity

There is concern that Indonesia would “collapse” if food diversification is not rapidly strengthened in the face of increasing public consumption of imported wheat. The figure for Indonesia's wheat import demand in 2019 was 10.69 tons; in 2020, it reached 10.29 tons; and in 2021, it increased to 11.17 tons [50]. The analysis results show that the wheat commodity has a negative value, indicating that it is a relatively soft good (P = −0.39 and I = −0.04) because rising income reduces demand for flour. This shows that wheat-based foods are not now causing a threat to the Indonesian people. Food local commodities are still the primary source of food. This is also evidenced by wheat's negative cross-elasticity to other foods (rice, corn, cassava, and sweet potatoes), implying that increasing other foods will reduce wheat consumption. As a result, the community continues to prefer local food for their needs (Table 5).

**Table 5:** Model of wheat demand function on the prices of other food commodities.

Commodities	Demand function model for wheat commodity to other crops	Demand elasticity Coefficient (Є)
Wheat	Q_wheat=_9.32 P ^−0.39^_wheat_ I^−0.04^_wheat_	ЄP_wheat_ = −0.39; ЄI_wheat_ = −0.04
Rice	Q_wheat_ = 12.60 P^-0.76^_rice_	−0.76**
Corn	Q_wheat_ = 12.71 P^-0.85^_corn_	−0.85**
Cassava	Q_wheat_ = 6.83 P^-0.16^_cassava_	−0.16**
Sweet potato	Q_wheat_ = 6.97 P^-0.17^_sweet potato_	−0.17**

Notes: * = significant effect at α level of 10%.

** = significant effect at α level of 5%.

Several studies have shown that the nutritional content of local food is better than wheat, as traditional tuber flour's physicochemical contents are better than wheat flour. Another aspect, wheat and corn production will be increasingly impaired by ecological drivers such as land degradation, water scarcity and climate change [22,56].

## 5 The dynamics of income to the pattern of community demand

Many factors determine the community's food consumption pattern, but the two primary ones are income and the community's knowledge of food and nutrition. The analysis results show that the level of community welfare continues to rise, as evidenced by an increase in people's income. However, changes in income have not had a major qualitative impact on changes in consumption patterns. In certain ways, changes in people's income have not changed food consumption patterns that positively impact health and improve the quality of human resources. Food preferences change regularly. Price changes will have an impact on preferences. According to the law of demand, customers will reduce the consumption of commodities when the prices rise and vice versa. Income has an impact on preferences as well. Households with higher earnings will have a higher preference for carbohydrate-rich foods compared to low-income households. Due to their limited resources, low-income households may not have many options for changing their food patterns [23,57].

The corn commodity is of concern because the elasticity value is very significant and the largest among other food commodities >1 (Table 6). Currently, the corn-based processed food and feed industries are growing rapidly. In addition, people affected by health problems switch to consuming corn as staple food. Corn, as a functional food, contains much dietary fiber needed by the body, essential fatty acids, isoflavones, minerals (Ca, Mg, K, Na, P, Ca and Fe), anthocyanins, beta-carotene (provitamin A), essential amino acid composition, and others [58].

**Table 6:** Matrix of demand elasticity for food commodities.

Commodities	Elasticity of price					Income elasticity of demand
	Rice	Corn	Cassava	Sweat potato	Wheat	
Rice	0.26*	1.03**	0.16**	0.18**	0.58**	0.24**
Corn	3.10**	5.09*	1.00**	1.03**	2.81**	0.13
Cassava	2.84**	4.31**	0.62**	1.00**	2.73**	0.51
Sweat potato	0.54*	1.21**	0.19**	0.16	1.00	0.15
Wheat	−0.76**	−0.85**	−0.16**	−0.17**	−0.39	−0.04

Notes: * = significant effect at α level of 10%.

** = significant effect at α level of 5%.

Income elasticity (ЄI) measures the sensitivity of demand for carbohydrate food sources to income. The dynamics of increasing income for cassava is greater than for other foods in influencing the demand (ЄI cassava = 0.51) (Table 6). This shows that cassava has an opportunity to become a prospective food in the future related to the dynamics of people's income. Cassava has a low glycemic index (GI), making it suitable for people with diabetes. Cassava can substitute rice as an alternative food source in Indonesia [45,59]. A decrease in income reduces wheat flour consumption; vice versa, an increase in income increases wheat flour consumption. However, the increase is smaller than local food commodities and insignificant. In conclusion, the data in Table 6 reveal that the concern that wheat dominance shifts local food is unjustified for the time being.

### 5.1 The policy implication

1. Global issues in the future

Currently, rice provides 96% of the calorie and protein needs of the Indonesian population and 70% of most of the Asian population, especially the poor. Rice surpasses other foods in terms of nutrition. Rice is edible in all parts and has 360 calories per 100 g and 6.8 g of protein per 100 g. Rice contributed to 54.3% of per capita energy consumption. That is, rice makes up more than half of our energy intake. Rice supplies about 40% of the protein in Asian [60,61].

Rice farming in Indonesia supports 25.4 million households or more than half of the country's population. In short, rice is a vital commodity for Indonesia since it contributes to its triple security: food, economic, and national. Most Asian countries are particularly interested in rice, not just as a wage good but also as a political commodity. Rice is an important economic commodity in Asia. It is not surprising that these countries allocate additional sums for farmers, both through various subsidy schemes and the construction of dams and irrigation networks, because rice farming is still applied by millions of farmers in most countries, including Vietnam, Myanmar, Thailand, India, and China, and rice contributes to the country's foreign exchange [61].

During 1996–1998, China allocated USD 18.2 billion per year for green box policies in the agriculture sector [62]. India provides subsidies for fertilizers, fuel, agricultural equipment, and various output pricing policies [63]. In addition to providing export credit subsidies and collateral-free bank loans, Thailand created a paddy mortgage scheme through the Bank of Agriculture and Cooperatives [64]. Everything is done with the main goal of meeting household food demands (self-sufficiency).

## 6 Anticipatory strategic steps for food in Indonesia

Indonesia's agricultural land has enormous potential in the agriculture sector. There are 100.7 million ha appropriate for agricultural land, of which 24.5 million ha are good for wetland (rice fields), 25.3 million ha are suitable for dry land for seasonal crops, and 50.9 million ha for dry land for annual crops. Because of its tropical environment, Indonesia permits agricultural business to be done annually [64,65].

The following action plans should be taken to anticipate the global food crisis:

### 6.1 Increasing national food reserves

The government stabilizes the supply and pricing of staple foods, particularly rice, to maintain farmers' income and purchasing power while maintaining consumer affordability. One of the stabilization efforts is the management and maintenance of the Government Rice Reserves (CBP). The fundamental reason for considering the importance of creating national rice reserves is the global rice market's volatility and instability [66]. According to the data, the global rice trade volume is small, reaching only 10% of the total global rice output [67].

Total rice production increased between 2000 and 2016, according to data from the United States Department of Agriculture (USDA). However, the ratio of rice exports to rice production did not increase significantly (about 10% of total production), indicating that the international rice market was relatively low. This can be an obstacle, especially during a food crisis. For example, many countries implemented export restrictions during the global food crisis of 2007/2008. Among them include raising export taxes and limiting the amount of rice exported. These limits are implemented not just by rice-exporting countries but also by rice-importing and re-exporting countries. In this context, the capacity of a country to reduce the need for consumer goods, particularly during a crisis, must be increased [[68], [69], [70]].

Food reserves are one of the price stability instruments, particularly for overcoming seasonal food production patterns and anticipating the consequences of international market shocks. As mandated by Food Law 18/2012, Indonesia has established a multilayered mechanism of national food reserves, consisting of a central government food reserve, regional government food reserves (provincial, district/city, and village level), and community food reserves [71]. According to Presidential Decree Number 48 of 2016 concerning Assignments to Indonesian National Logistics Agency (*Bulog)* in the Framework of National Food Security, it is stipulated that *Bulog* is assigned to manage Government Rice Reserves (CBP) in order to maintain food availability and stabilize food prices at the consumer and producer levels for the staple food type rice. The amount of government rice reserves is determined regularly, keeping into account the level of community needs.

There are three main methods for calculating CBP. First, the Food and Agriculture Organization (FAO) uses the concept of Stock Utilization Ratio (SUR), which is the ratio of rice stock/supply to total population rice needs (consumption and other needs). FAO recommends a safe SUR figure of 17–18% [61]. Second, the ASEAN Food Security for Information System (AFSIS) study recommends that national rice reserves represent 20% of the total national rice demand. Third, ASEAN countries were compared at the Policy Workshop on Food Security and Disaster Risk Reduction in Asia in 2018, held in Bangkok, Thailand. The amount of CBP can be determined by calculating the national need for rice for the entire population of a country facing an emergency within a specific time frame. CBP plays a strategic role in maintaining rice price stability, dealing with emergencies, disasters, and food insecurity, implementing the ASEAN Plus Three Emergency Rice Reserves Agreement, international cooperation in social assistance, and other needs that are in the best interests of the government [72]. CBP, controlled by *Bulog*, is primarily responsible for managing food prices through market operations and social or natural disaster emergency assistance [73].

*Bulog* will play two roles in increasing national food reserves, according to food policy. First, *Bulog* has a role as an operator for food procurement and price stabilization must be reinforced. Second, *Bulog* ensures food security, including price support for farmers, keeping prices accessible for consumers, and delivering food help to those who need it.

### 6.2 Functional food diversification

Food diversification is difficult to achieve quickly since it is strongly related to a preference (‘taste’) and consumption habits. The general people have consumed rice for a long time, and with food diversification, this will drastically change to cassava, sweet potatoes, or corn, for example. In Indonesia, there is a lack of socialization and promotion of the importance and value of these non-rice carbohydrate food sources.

Along with the increasing public awareness on the importance of healthy living, consumer food preferences are also changing. Foods that are becoming increasingly popular among consumers must not only have a high nutrient quality and an appealing appearance and taste, but they must also fulfill certain physiological functions for the body. Specifically, food is functional food or healthy food, so eating does not only fill the stomach but also improves body health and fitness.

Despite high calories (approximately 123 calories per serving), sweet potatoes also have high concentration of nutrients, particularly red sweet potatoes. Red sweet potatoes have higher vitamin A concentration than grains and tubers, up to 7,700 Sl. Sweet potato leaves contain more vitamin C than other fresh vegetables. It can reach 45–62 mg in sweet potato leaves but only about 25 mg in cassava shoots. Beta carotene and anthocyanins benefit health, particularly in preventing degenerative diseases such as coronary heart disease, stroke, and cancer [74,75].

### 6.3 Technological support in food production and processing

We are highly advanced in the field of sweet potato research and development. Since 1977, 14 superior and high-productivity varieties, including Daya, Prambanan, Borobudur, Mendut, Kalasan, Sukuh, Papua Solossa, Papua Pattipi, Sawentar, purple and yellow sweet potatoes, and so on, have been developed by the Indonesian Ministry of Agriculture. Cassava commodities from superior varieties of Adira 1, Adira 4, Malang 1, Malang 3, Malang 4, Malang 6, UJ-3, UJ-5, and so on have their own advantages, both in cultivation and in its utilization for industry and consumption. Similarly, food technology is already accessible, such as processing technology for sweet potatoes and cassava, for example chips, flour as a basic ingredient in various food products such as breads, cakes, and so on [76].

Non-rice and non-wheat processing technology development are limited. Rice and wheat flour are readily available in the market; however, flour from corn, cassava, sweet potato canna, and taro is available in limited quantities and not permanently. In addition, when compared to rice and wheat, processing technology, including equipment for local food, has not been optimally developed, because rice cookers are well known but there are no corn or cassava cookers yet. Even if there is, the development, dissemination, and absorption of local food processing technology to improve processing practicality, nutritional value, economic value, social value, image, and acceptance are slow. Even non-rice alternative food industries made from tubers, such as instant traditional *tiwul* and *gatot* from cassava, casava flour, *bija flour* (from sweet potato), and so on, are still relatively limited and even on a small or domestic scale. The development of these types of industries is quite slow. Many factors influence it aside from the issue of raw material supply continuity and marketing concerns related to limited demand. Food diversification education and promoting nutritious foods are important for the community in advancing food diversification.

Food diversification patterns and improved nutritional fulfillment can be carried out by formal and non-formal institutions in the community. Understanding of healthy food must be done from an early age. Example of non-formal institutions in the community is health activities for mothers and children such as supplementary feeding activities (PMT). The activity's target is the attainment of the expected food pattern towards diverse, nutritious, and balanced food. The level of acceptance and resistance to non-rice food innovations in food security is still difficult for the public to accept, because non-rice carbohydrate foods only have the status of snacks.

### 6.4 Preference and pride of local food products

The public is still unaware of the importance of food diversification and nutrition. The prestige factor is sometimes more dominant than the health aspect in eating patterns, sometimes acting impulsively. This includes raising public knowledge about food safety. Formal and non-formal institutions like integrated service institutions (*Posyandu*) can carry out food diversification and nutritional fulfillment patterns. This institution is involved with local community health activities, particularly those that involve mothers and children, such as supplementary feeding programs. This activity aims to achieve the intended food pattern of diversified, nutritious, and balanced food. Non-rice alternative food programs are still lacking in this activity. Because non-rice carbohydrate foods are solely considered snacks, the public's acceptance, and resistance to non-rice food improvements in security remains difficult.

## 7 Conclusion

Based on the price elasticity of demand, rice, maize, cassava, and sweet potato commodities are inelastic, meaning they are not responsive to changes in its price. The cross elasticity of these non-wheat commodities shows mutual substitution between the carbohydrate source foods. This is indicated by cross elasticity >0. The four non-wheat food commodities are normal goods. An increase in income, for example, will also increase consumption. The maize commodity is of concern because the demand elasticity value is significant and the largest among other food commodities >1. At the same time, the dynamics of increasing income for cassava is greater than for other foods in influencing the demand. This indicates that maize and tubers are prospectively for food in the future. The dynamics of an increase in income will reduce the demand for wheat (I negative) for all food commodities, and the decrease in the effect will not be significant. This means that wheat food is only a complement, not a staple food need, so the concern about the domination of wheat as a food ingredient with practically processed products in society has not shifted local food significantly. Facing the global food crisis, the anticipatory steps are the availability of high yielding varieties of rice, corn, cassava, and sweet potatoes, implementing food reserves by *Bulog* from the center to the government regions, food diversification, changing preferences to consume local food with pride.

## Author contributions

Conceived and designed the experiment: F Rozi, A B Santoso, V Siagian, H Subagio, and A Syam; Performed the experiments: F Rozi, A B Santoso, I G A P Mahendri, R T P Hutapea, D Wamaer, V Siagian, D A A Elisabeth, H Handoko, S Sugiyono, H Subagio, and A Syam; Analyzed and interpreted the data: F Rozi, A B Santoso, R T P Hutapea, D Wamaer, V Siagian, H Handoko, S Sugiyono, H Subagio, and A Syam; Contributed materials, analysis tools, or data: I G A P Mahendri, R T P Hutapea, D Wamaer, D A A Elisabeth, H Handoko, and S Sugiyono; Wrote the paper: F Rozi, I G A P Mahendri, V Siagian, D A A Elisabeth, H Subagio, and A Syam. All authors have read and agreed to the published version of the manuscript

## Funding

This research was funded by National Research and Innovation Agency Republic of Indonesia - BRIN.

## Data availability statement

The datasets generated during and/or analyzed during the current study are available at the following link: https://data.mendeley.com/datasets/n6yfrp8gf3/1.

## Declaration of competing interest

The authors declare that they have no known competing financial interests or personal relationships that could have appeared to influence the work reported in this paper.
